# Effect of Baduanjin exercise on acute myocardial infarction in patients with anxiety and depression after percutaneous coronary intervention: A randomized controlled trial

**DOI:** 10.1097/MD.0000000000040225

**Published:** 2024-11-08

**Authors:** Liang Kang, Yihua Li, Keyu Chen, Xiaoqin Chen, Qingmin Chu, Xinjun Zhao, Rong Li

**Affiliations:** a The First Affiliated Hospital of Hebei North University, Zhangjiakou, Hebei Province, China; b The First Clinical Medical College of Guangzhou University of Chinese Medicine, Guangzhou, China; c Department of Interventional Room, The First Affiliated Hospital of Guangzhou University of Chinese Medicine, Guangzhou, China; d Department of Cardiovascular Disease, The First Affiliated Hospital of Guangzhou University of Chinese Medicine, Guangzhou, China.

**Keywords:** exercise tolerance, myocardial infarction, percutaneous coronary intervention, quality of life

## Abstract

**Background:**

Acute myocardial infarction (AMI) is the most severe type of coronary heart disease and patients often require percutaneous coronary intervention (PCI). However, anxiety and depression are common complications of PCI.

**Methods:**

This study conducted a prospective randomized controlled trial to investigate the effectiveness of Baduanjin exercise in improving cardiac function and alleviating symptoms of anxiety and depression in patients who underwent PCI for AMI. Patients with AMI (n = 120) who underwent PCI at the Cardiology Department of The First Affiliated Hospital of Guangzhou University of Chinese Medicine between September 2020 and June 2021 were included. Participants were divided into a control group (moderate intensity walking) and a Baduanjin exercise group. The treatment period was 8 weeks, with follow-ups at weeks 4 and 8. Improvement of cardiac structure and function indices was measured by echocardiography. Physical function was assessed using the 6-minute walk test. Mental state was assessed using the Seattle Angina Questionnaire, Hamilton Anxiety Scale, and Hamilton Depression Scale before and after exercise.

**Results:**

Compared to the control group, the Baduanjin exercise group showed improvement in change in left ventricular end-diastolic diameter (–1.0 ± 3.4 vs –2.3 ± 2.7 mm, respectively; *P* = .02), left ventricular ejection fraction (6.8 ± 4.2 vs 4.5 ± 4.3%, respectively; *P* = .002), angina stability (42.5 ± 31.7 vs 33.3 ± 29.7, respectively; *P* = .11), 6-minute walk test (118.4 ± 49.1 vs 88.3 ± 40.2 m, respectively; *P* < .001), and Hamilton Anxiety Scale (6.7 ± 2.6 vs 5.3 ± 2.6, respectively; *P* = .003), and Hamilton Depression Scale (7.6 ± 4.1 vs 4.8 ± 2.1, respectively; *P* < .001) scores.

**Conclusions:**

Baduanjin exercise for 8 weeks improved the cardiac function and mental state of patients with anxiety and depression after PCI for AMI. Through this study, we aim to provide reliable evidence in support of the beneficial effects of Baduanjin exercise on cardiac function and anxiety–depression, contributing to evidence-based medicine.

## 1. Introduction

Acute myocardial infarction (AMI) is a coronary artery disease (CAD) characterized by a sharp reduction in coronary artery blood supply, causing acute necrosis of the corresponding portion of the myocardium due to severe persistent ischemia. AMI negatively impacts quality of life and life expectancy and imposes a heavy financial burden on patients and their families.^[1–3]^ AMI is also a worldwide epidemic, and the number of patients in China is significant and increasing annually.^[4]^ Treatment of AMI includes thrombolysis therapy, coronary artery bypass grafting, and percutaneous coronary intervention (PCI). PCI is the current preferred clinical measure, which promptly relieves coronary artery stenosis, restores myocardial perfusion, and reduces the mortality of patients with AMI.^[5]^ PCI has a high safety profile with few side effects and complications.^[6]^

Wang et al^[7]^ showed that patients with CAD who also experience anxiety and depression had a higher incidence of major adverse cardiovascular events. Anxiety and depression after PCI can increase the risk of recurrent major adverse cardiovascular events by 2 to 3 times, affecting quality of life and long-term prognosis and increasing the risk of death in the 10 years following PCI by 77%.^[8]^ Luttik et al^[9]^ also found that, among patients with coronary heart disease (CHD), 42% and 26% also had depression and anxiety, respectively. Moreover, 20% of patients had mood disorders in the middle and late stages of treatment. Similarly, Tully et al^[10]^ found that 20% of patients with CHD had emotional disorders, and 40% had anxiety and depression. This demonstrates that anxiety and depression after myocardial infarction should receive more clinical attention.

Baduanjin is a traditional exercise method that is practiced widely, with significant influence in China. It has been extensively used in rehabilitation medicine with the clinical effects of improving cardiopulmonary function and exercise tolerance and reducing the incidence of adverse events.^[11–13]^ However, evidence-based studies investigating the effect of Baduanjin exercise on cardiac function and clinical symptoms in patients with anxiety and depression after PCI are limited. Therefore, this study aimed to evaluate the effect of Baduanjin exercise on cardiac function, exercise tolerance, mental state, clinical symptoms, and quality of life in patients with AMI and anxiety and depression after PCI.

## 2. Methods

### 2.1. Ethical experimentation

This randomized controlled trial was approved by the Ethics Committee of The First Affiliated Hospital of Guangzhou University of Chinese Medicine, Guangzhou, China (approval number: ZYYECK [2020] 045; approval date: May 08, 2020). All procedures performed in studies involving human participants were in accordance with the ethical standards of the institutional and/or national research committee and with the 1964 Declaration of Helsinki and its later amendments or comparable ethical standards. The study was conducted and reported in accordance with the Consolidating Standards of Reporting Trials (CONSORT) 2010 statement. The study was registered with the Chinese Clinical Trial Registry (ClinicalTrials.gov: ChiCTR2100047298; registration date: November 06, 2021). All participants provided written informed consent for inclusion in the study. The trial protocol has been published.^[14]^

### 2.2. Participants

Patients aged 18 to 75 years, admitted to the Cardiology Department of The First Affiliated Hospital of Guangzhou University of Chinese Medicine, Guangzhou, China, between September 2020 and June 2021, with a diagnosis of AMI who underwent PCI were evaluated based on the inclusion and exclusion criteria. The diagnosis of acute ST-segment elevation myocardial infarction was made based on the Chinese Society of Cardiology of Chinese Medical Association 2019 guidelines.^[15]^ Similarly, acute non-ST-segment elevation myocardial infarction was defined according to the European Society of Cardiology 2020 guidelines.^[16]^ Patients with Killip Grade I–II cardiac function, moderate- to low-risk exercise rehabilitation risk stratification after PCI,^[17]^ anxiety and depression, or a tendency towards anxiety and depression (Hamilton Anxiety Scale [HAMA] and/or Hamilton Depression Scale [HAMD] score ≥ 3), smooth gait, and consciousness were enrolled. The exclusion criteria were as follows: high-risk post-PCI exercise rehabilitation risk stratification or restricted activity for other reasons,^[17]^ poorly controlled hypertension (systolic blood pressure ≥ 180 mm Hg and/or diastolic blood pressure ≥ 100 mm Hg), severe renal insufficiency, hepatic insufficiency, severe chronic obstructive pulmonary disease, cor pulmonale or respiratory failure, hematopoietic system disease, cancer, other serious primary diseases, and participation in methods of traditional Chinese medicine (Tai Chi, Baduanjin, etc) or other clinical trials for approximately 3 months. In total, 120 eligible patients were enrolled.

### 2.3. Randomization and masking

**Figure 1.**
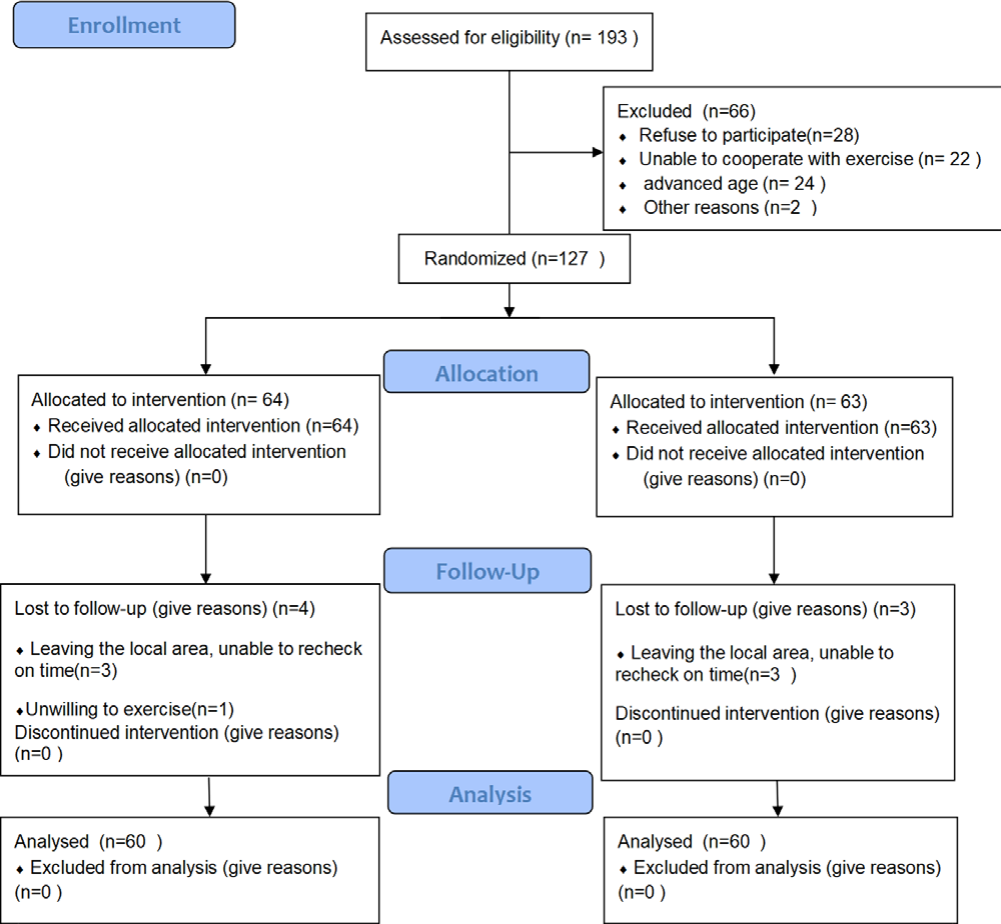
Flow diagram of the clinical trial design. Flow of patients through the study. Of 193 patients who were screened, 120 were considered eligible and were randomly assigned to the Baduanjin exercise or control group. In both groups, 60 patients completed the study.

Patients who met the inclusion criteria were block randomized to 1 of 2 groups: the Baduanjin exercise (A) and control (B) groups. The blocks (AABB, ABAB, ABBA, BAAB, BABA, and BBAA) were numbered, and a random-number generator was used to randomly assign 60 patients to each group. Group assignments were sealed in opaque envelopes, and randomization was performed by a statistician. Given the variability in exercise prescriptions, it was not possible to blind the investigators and study participants to the group allocations. However, the echocardiographers, 6-minute walk test (6-MWT) assessors, and statisticians were blinded to the group allocations. The study recruitment process is outlined in Figure 1.

### 2.4. Sample size

The study protocol for this study has been publicly disseminated.^[14]^ In accordance with the established study protocol, PASS 15 software was employed to calculate the required sample size. Considering a significance level (type-I error rate) of *α* = 0.05, a desired statistical power of 80% (type-II error rate of *β* = 0.2), and accounting for a potential dropout rate of 10%, it was determined that a minimum of 57 patients per group (experimental and control) was necessary.

### 2.5. Interventions

Patients in both groups were administered a standardized treatment regimen for secondary prevention of CHD combined with routine patient education. Drugs included antiplatelet aggregators, angiotensin-converting enzyme inhibitors/angiotensin receptor blockers/angiotensin receptor–neprilysin inhibitors, lipid-regulating drugs, and β-blockers. The patients’ psychological status was monitored remotely via WeChat. antianxiety and depression drugs were temporarily excluded during the study period.

Cardiac rehabilitation exercises consisted of 2 phases in both groups. Phase I included the CHD Care Unit and general ward phase, specifically referring to the flow diagram of exercise rehabilitation after PCI and the rehabilitation program for intermediate- and high-risk patients after PCI for 1 week.^[17]^ Routine rehabilitation training was performed under the guidance of the investigator 2 to 5 days after PCI. Walking exercise and Baduanjin learning and preliminary practice began 6 to 7 days after surgery (the average length of hospital stay after PCI for AMI was 7 days). According to the evaluation of the standing movement of patients (see Figure S1, Supplemental Digital Content, http://links.lww.com/MD/N794, which shows the orthostatic exercise test), slow walking training was performed in the ward with selective learning and practice of Baduanjin once a day with 1 or 2 attempts (each attempt lasted 15 minutes). Based on the mean duration of hospitalization among the participants, Phase I typically spans approximately 7 days.

Phase II included the outpatient rehabilitation or early out-of-hospital rehabilitation phase. The participants initiated self-directed home-based rehabilitation training starting from the day immediately following their discharge. This training was carried out under the supervision of trainers through a WeChat group. Additionally, the participants also underwent outpatient rehabilitation training during their follow-up visits. Participants in the control group performed moderate intensity walking exercise on a treadmill or outdoors 5 times per week for 8 weeks. Each exercise consisted of 3 components: a 5-minute warm-up, at least 20 minutes of walking, and 5 minutes of relaxation. Resting heart rate was recorded before exercise, and exercise intensity was controlled at approximately 20 bpm higher than the resting heart rate^[18]^ (The heart rate monitoring was performed utilizing the W1M smartwatch, developed by Lingmeng Technology Co., Ltd. in Shenzhen, China.), or according to the patient’s perception of little effort (Borg score: 0.5–1) (see Table S1, Supplemental Digital Content, http://links.lww.com/MD/N795, which shows the Borg scale). Records were made on the patient exercise record sheet (see Table S2, Supplemental Digital Content, http://links.lww.com/MD/N795, which shows the patient exercise record sheet). All participants were instructed to maintain their usual activities and to avoid new strength training. Phase II has a duration of 8 weeks in total.

Patients in the Baduanjin exercise group practiced Baduanjin independently for 5 days per week, once in the morning and once in the evening every day (records were made on the patient exercise record sheet). The duration of each practice was approximately 15 minutes, with a total daily duration of approximately 30 minutes. The observation period was 8 weeks. Each exercise was divided into 3 steps, namely, the preparation, activity, and recovery periods. The preparation period generally lasted 10 minutes, including preparation of the body and relaxation of the mind and spirit. The recovery period lasted approximately 5 minutes, including stretching exercises of the upper and lower limbs, balance training, such as standing on 1 foot, coordination training, and breathing adjustment. Exercise time depended on the patient’s physical strength; however, 15 minutes was generally recommended. Exercise intensity was briefly assessed using the target heart rate method or Borg scale.^[17]^ Resting heart rate was recorded before exercise, and exercise intensity was controlled at approximately 20 bpm higher than the resting heart rate^[18]^ (The heart rate monitoring was performed utilizing the W1M smartwatch, developed by Lingmeng Technology Co., Ltd. in Shenzhen, China.), or according to the patient’s perception of little effort (Borg score: 0.5–1) (see Table S1, Supplemental Digital Content, http://links.lww.com/MD/N795, which shows the Borg scale). If the increase in heart rate after exercise or daily activities was >20 bpm and the patient felt strained, the exercise intensity was adjusted and reevaluated. The Baduanjin training video (available from: https://v.youku.com/v_show/id_XNDc1MTU2MzEwMA==) was uploaded for patient follow-up. The Baduanjin exercise program included 8 forms (approximately 15 minutes each): form 1, propping up the sky; form 2, drawing the bow; form 3, raising 1 hand; form 4, looking over the shoulders; form 5, clenching fists and looking forward with eyes wide open; form 6, pulling toes; form 7, swaying the head and buttocks; and form 8, jolting.

In this study, we employed WeChat groups for the purpose of guiding and supervising the daily rehabilitation exercises of subjects post-discharge. Additionally, we mandated that subjects complete the patient exercise record sheet (see Table S2, Supplemental Digital Content, http://links.lww.com/MD/N795) and reviewed these records during each subsequent outpatient follow-up appointment to ensure adherence to rehabilitation exercises outside of the hospital.

### 2.6. Outcomes

The primary outcomes assessed were the changes in left ventricular ejection fraction (ΔLVEF) and left ventricular end-diastolic diameter (ΔLVEDD) using echocardiography. These measurements were carried out by trained physicians following a standardized protocol. Secondary outcomes included the 6-MWT and HAMA, HAMD, and Seattle Angina Questionnaire (SAQ) scores. For the 6-MWT, the 6-MWT distance and Borg scale were measured. Patients were asked to walk back and forth along a 100-m long flat straight road for 6 minutes, and the total distance walked was calculated. The Borg scale was used to evaluate patients’ fatigue before and after exercise. For HAMA, the total score was calculated and interpreted as follows: ≥29 (severe anxiety), ≥21 (obvious anxiety), ≥14 (anxiety), >7 (mild anxiety), and <7 (no anxiety). For HAMD, the score correlated with disease severity. A total HAMD score of <7 denoted normal; 7 to 17, possible depression; 17 to 24, definite depression; and >24, severe depression. In the SAQ, the standard score [(actual score − lowest score)/(highest score − lowest score) × 100] correlated with quality of life and physical function.

The primary and secondary outcome measures of participants in both the Baduanjin exercise group and the control group were evaluated during the washout period (7 days before enrollment) and at 8 weeks posttreatment, excluding 6-MWT. The 6-MWT distance was examined at the fourth- and eighth-week of treatment. The evaluation program and follow-up plan for the control group were the same as those for the experimental group. Patients were telephoned to visit the hospital’s outpatient clinic for follow-up. Researchers conducted detailed telephone follow-ups for patients who could not visit the clinic or be followed up in a timely manner.

### 2.7. Statistical analysis

The statistical analysis in this study adheres to the intention-to-treat principle. The percentage of missing values for the variables among the subjects under follow-up is <5%. Data are expressed as means ± SD. Two independent sample *t* tests were used when the data followed a normal distribution, and the variance was homogeneous. Tamhane T2 test was used when the variance was heterogeneous. Chi-square and Wilcoxon rank sum tests were used for count and rank data, respectively. All statistical analyses were conducted using SPSS 22.0 (IBM Corp., Armonk, NY). A *P* < .05 was considered significant.

## 3. Results

**Figure 2.**
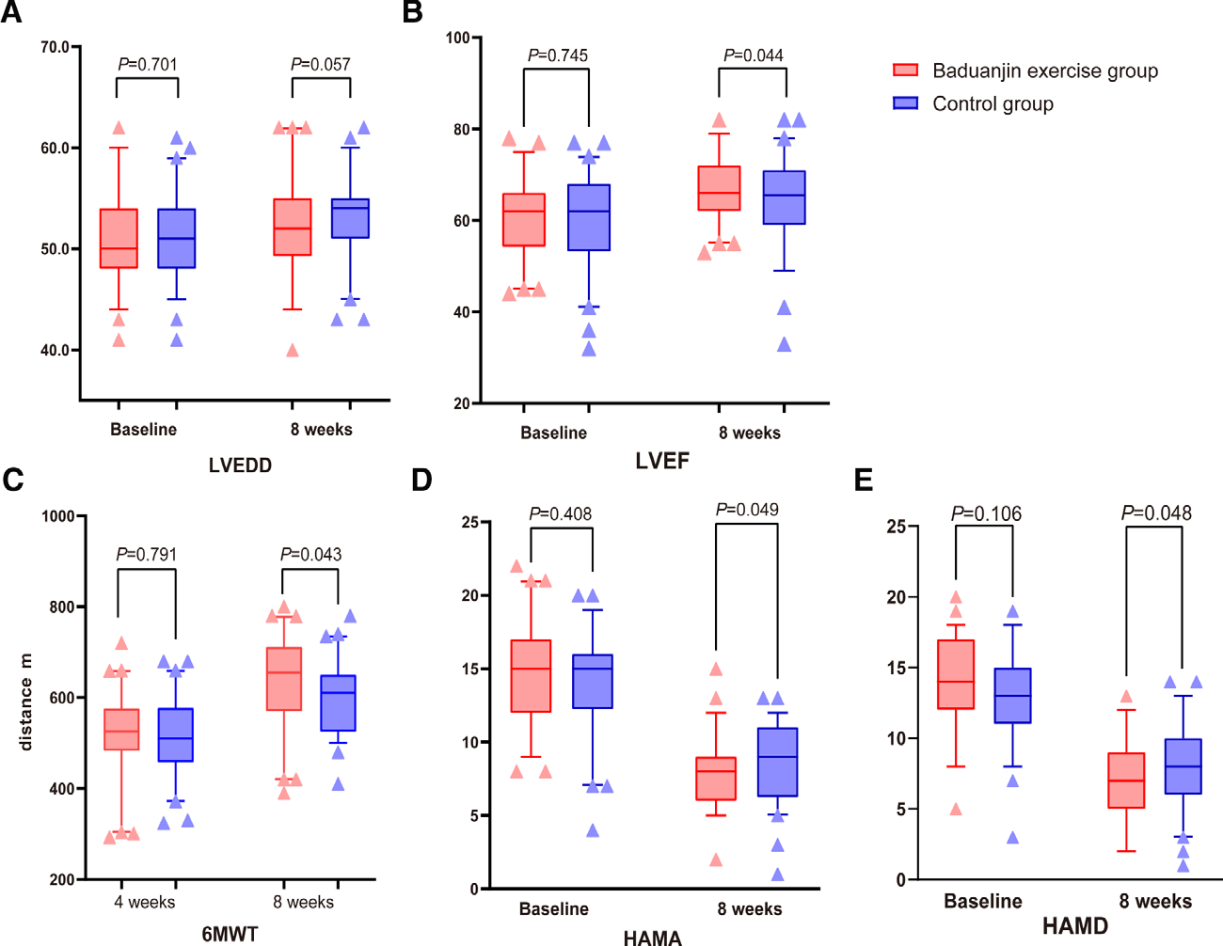
Comparison of baseline and follow-up outcome indicators. Comparison of (A) LVEDD and (B) LVEF between the 2 groups at baseline and after 8 weeks of treatment. Comparison of (C) 6-MWT between the 2 groups after 4 and 8 weeks of treatment. Comparison of (D) HAMA and (E) HAMD scores between the 2 groups at baseline and after 8 weeks of treatment. 6-MWT = 6-minute walk test, HAMA = Hamilton Anxiety Scale, HAMD = Hamilton Depression Scale, LVEDD = left ventricular end-diastolic diameter, LVEF = left ventricular ejection fraction.

Baseline demographic data were comparable between the 2 groups, with no significant differences in age, sex, myocardial infarction type, culprit vessel, risk factors, and baseline medication (Table 1). There was no significant difference in LVEDD between the 2 groups before treatment. However, LVEDD increased slightly in both groups after treatment, although the difference was not significant (Fig. 2A). The differences before and after treatment showed that the Baduanjin exercise group had a smaller ΔLVEDD than the control group (–1.0 ± 3.4 vs –2.3 ± 2.7 mm, respectively; *P* = .02). There was no significant difference in LVEF between the 2 groups before treatment. Although LVEF significantly increased in both groups after treatment, a larger ΔLVEF was observed in the Baduanjin exercise group, with a significant difference (6.8 ± 4.2 vs 4.5 ± 4.3%, respectively; *P* = .002) (Fig. 2B).

**Table 1:** Baseline characteristics of patients in the Baduanjin exercise and control groups.

	Baduanjin exercise group (n = 60)	Control group (n* *= 60)	*P*-value
Sex (male/female)	57/3	56/4	.70
Age, years (mean ± SD)	52.2 ± 10.9	53.0 ± 10.7	.67
AMI classification [n (%)]			
STEMI	46 (76.7)	49 (81.7)	.50
NSTEMI	14 (23.3)	11 (18.3)	
Culprit vessel [n (%)]			
Left anterior descending artery	29 (48.3)	28 (43.3)	>.99
Left circumflex artery	12 (20)	15 (25)	.66
Right coronary artery	22 (36.7)	21 (35)	>.99
Risk factor [n (%)]			
Hypertension	25 (41.7)	27 (45.0)	.71
Type 2 diabetes mellitus	25 (41.7)	25 (41.7)	>.99
Dyslipidemia	35 (58.3)	39 (65.0)	.57
Smoking	44 (73.3)	42 (70.0)	.84
Overweight (BMI)	46 (76.7)	46 (76.7)	>.99
Medications [n (%)]			
DAPT	60 (1)	60 (1)	>.99
β-blockers	60 (1)	59 (98.3)	>.99
Statins	60 (1)	60 (1)	>.99
ACEI/ARB/ARNI	56 (93.3)	58 (96.7)	.68
Organic nitrates	6 (10.0)	8 (13.3)	.78
Out-of-hospital exercise compliance[n (%)]*	40 (66.7)	42 (70.0)	.70

ACEI = angiotensin-converting enzyme inhibitor, AMI = acute myocardial infarction, ARB = angiotensin receptor blocker, ARNI = angiotensin receptor–neprilysin inhibitor, BMI = body mass index, DAPT = dual antiplatelet therapy, NSTEMI = non-ST-segment elevation myocardial infarction, STEMI = ST-segment elevation myocardial infarction.

* The statistical data comprises the count of subjects who maintained a minimum of 4 days of weekly rehabilitation exercise training throughout the 8-week intervention period.

The 6-MWT was not performed during the run-in period because the condition of the patients with AMI was still unstable after primary PCI, and patients were still in the high-risk phase of the perioperative period. Therefore, the 6-MWT distance measured at the fourth- and eighth-week visits was compared in this trial. There was no significant difference in the 6-MWT between the Baduanjin exercise and control groups after 4 weeks of treatment. The walking distances of patients in both groups increased after 8 weeks of treatment, with a greater increase in the Baduanjin exercise group. The difference between the 2 groups was significant (118.4 ± 49.1 vs 88.3 ± 40.2 m, respectively; *P* < .001) (Fig. 2C).

Before treatment, there was no significant difference in anxiety levels and HAMA scores between the Baduanjin exercise and control groups. After treatment, HAMA scores decreased in both groups, with a greater decrease in the Baduanjin exercise group (6.7 ± 2.6 vs 5.3 ± 2.6, respectively; *P* = .003) (Fig. 2D). Before treatment, there was no significant difference in HAMD scores between the 2 groups. After treatment, HAMD scores decreased in both groups, with a greater decrease in the Baduanjin exercise group (7.6 ± 4.1 vs 4.8 ± 2.1, respectively; *P* < .001) (Fig. 2E).

Before treatment, there was no significant difference in the 5 aspects of the SAQ scores between the Baduanjin exercise and control groups. After 8 weeks of cardiac rehabilitation, both groups showed improvement in physical limitation, angina stability, angina frequency, treatment satisfaction, and disease cognition. Angina stability was better in the Baduanjin exercise group than in the control group, and the difference was significant (*P* = .036). There were no significant differences in other indicators between the 2 groups (Table 2).

**Table 2:** Comparison of Seattle Angina Questionnaire scores between the Baduanjin exercise and control groups.

	Group	Baseline	8 weeks	*P*-value	△*
Physical limitation	Baduanjin exercise group (n = 60)	56.6 ± 12.2	65.5 ± 11.0	<.001	8.9 ± 6.8
	Control group (n = 60)	60.0 ± 7.8	62.3 ± 8.3	<.001	2.3 ± 4.1
	*P*-value	.07	.08		.001
Angina stability	Baduanjin exercise group (n = 60)	27.9 ± 21.1	70.4 ± 19.3	<.001	42.5 ± 31.7
	Control group (n = 60)	30.4 ± 20.1	63.8 ± 17.5	<.001	33.3 ± 29.7
	*P*-value	.51	.05		.11
Angina frequency	Baduanjin exercise group (n = 60)	90.2 ± 11.7	96.0 ± 5.9	<.001	5.8 ± 8.5
	Control group (n = 60)	88.5 ± 11.8	93.8 ± 7.4	<.001	5.3 ± 9.5
	*P*-value	.44	.08		.76
Treatment satisfaction	Baduanjin exercise group (n = 60)	79.8 ± 8.8	83.6 ± 7.7	<.001	3.8 ± 4.8
	Control group (n = 60)	81.1 ± 7.9	83.8 ± 7.7	.001	2.7 ± 5.8
	*P*-value	.40	.89		.27
Disease cognition	Baduanjin exercise group (n = 60)	77.9 ± 9.1	80.4 ± 8.2	<.001	2.5 ± 4.4
	Control group (n = 60)	76.4 ± 7.1	78.9 ± 7.6	<.001	2.5 ± 4.4
	*P*-value	.31	.29		.99

*△ = 8 weeks-baseline.

There were no adverse events, such as all-cause mortality, recurrent AMI, heart failure, malignant arrhythmia, cardiogenic shock, stroke, and pulmonary embolism, in either group during the study period. No patient complained of obvious discomfort during follow-up. The electrocardiogram, blood analysis, liver and kidney function, electrolyte levels, and stool and urine analysis of patients in both groups did not indicate aggravation or recurrence.

## 4. Discussion

This study focused on patients with AMI and anxiety and depression after PCI. We found that Baduanjin exercise improved cardiac function and psychological status. Guo et al^[19]^ evaluated HAMA and HAMD scores within 1 day after PCI in 2028 patients and found that anxiety and depression rates were as high as 46.1% and 36.1%, respectively. Ouyang et al^[20]^ also reached a similar conclusion, with reported rates of anxiety and depression in patients treated with PCI 1 day after surgery at 33.7% and 35.8%, respectively. Overall, the high incidence of anxiety and depression after AMI should receive more clinical attention.

The beneficial effects of Baduanjin exercise on cardiac rehabilitation have been established. However, its potential to alleviate anxiety and depression in patients with AMI after PCI necessitates further investigation. Therefore, through this prospective randomized controlled study, our objective is to gather evidence within the framework of evidence-based medicine concerning the impact of Baduanjin exercise on cardiac function and the anxiety–depression status of patients with AMI after PCI. Additionally, we aim to enhance awareness among clinical practitioners regarding the prevalent issue of anxiety and depression post-AMI and explore potential effective treatment methods for addressing these conditions.

Previous studies confirmed that female sex, smoking, unemployment, low income, sedentary lifestyle, unhealthy eating habits, and low educational attainment are associated with anxiety and depression in patients with CAD.^[21,22]^ Anxiety and depression excite the sympathetic nervous system, impair endothelial function, promote the secretion of catecholamines, platelet hyperfunction, inflammatory response, and atherosclerosis, eventually inducing or aggravating cardiovascular disease by stimulating the hypothalamus–pituitary–adrenal axis.^[23,24]^

Mindfulness and physical exercise have been shown to improve the clinical symptoms of patients with CAD complicated by anxiety and depression.^[10,25]^ As a representative type of qi gong, the training process of Baduanjin includes not only physical exercise but also mental stability. Therefore, as an exercise prescription for cardiac rehabilitation, it has the potential advantage of exerting a comprehensive curative effect.

LVEF and LVEDD are common indicators of left heart function on echocardiography. After AMI, LVEF gradually decreased, whereas LVEDD increased, indicating a poor prognosis. Most drugs for secondary prevention of CHD can improve left ventricular function and delay ventricular remodeling. Although the LVEDD of patients in both groups increased after standardized secondary prevention treatment, the ΔLVEDD in the Baduanjin exercise group was bigger than that in the control group. However, longer periods of observation and follow-up are needed to further evaluate the effect of Baduanjin exercise on improving ventricular remodeling in patients with AMI after PCI.

During the 8-week follow-up, the LVEF recovery amplitude of patients in the Baduanjin exercise group was more pronounced than that in the control group, demonstrating that Baduanjin exercise can promote the recovery of left ventricular systolic function and help to improve early recovery of cardiac function in patients with AMI after PCI.

Moreover, the walking distance in both groups improved during follow-up, due to the cardiac rehabilitation exercise, with a greater improvement in the Baduanjin exercise group, suggesting that Baduanjin exercise is better than moderate intensity walking in improving the cardiopulmonary function and exercise ability of patients with AMI after PCI.

After 8 weeks of rehabilitation, the level of anxiety and depression decreased in both groups, demonstrating that proper rehabilitation may help patients to gradually recover physically and regain a social life. Anxiety and depression can be relieved to a certain extent using this approach; however, the degree of relief and whether it can be restored to the preoperative level remains unknown. Even a low degree of anxiety and depression could inflict harm and affect the recovery of the patient. Nevertheless, compared with the control group, Baduanjin exercise accelerated the improvement in the postoperative psychological state.

Kala et al^[26]^ showed that the incidence of anxiety and depression after PCI decreased in the short-term with relief of chest tightness, chest pain, and other symptoms; however, the incidence gradually increased in the following year due to the recurrence of symptoms and need for long-term medication. The follow-up period in this study was only 8 weeks after PCI; thus, longer follow-up is needed to understand whether the psychological state fluctuates again after exercise completion or the cessation of Baduanjin exercise.

Through proteomic analysis, Shuai Mao et al (2021) demonstrated that Baduanjin exercise induces changes in the expression of 80 proteins and regulates metabolic processes, immune processes, and extracellular matrix remodeling in patients after myocardial infarction.^[27]^ Specifically, interleukins and TGF-β have been confirmed to be associated with anxiety and depression.^[28]^ Moreover, research by Wan Mingyue (2022) and others has revealed that Baduanjin exercise can modify the structural plasticity of subregions, including the left subiculum, hippocampal-amygdala transition zone, right entorhinal area 1, and anterior subiculum, which are involved in cognitive regulation.^[29]^ However, there is still limited understanding of the mechanisms underlying the effects of Baduanjin exercise, necessitating further investigation into its potential for improving anxiety and depression.

Among patients with AMI after PCI, approximately 50% still suffer from varying degrees of chest tightness and pain.^[30]^ The main reasons for ischemic chest pain include stent thrombosis, side branch occlusion, slow flow, no-reflow, incomplete revascularization, vascular dissection or hematoma, stent restenosis, microcirculatory disorders, and coronary spasm. In contrast, nonischemic chest pain is caused by stent reaction, anxiety and depression, and post-myocardial infarction syndrome.^[31]^ Therefore, the improvement effect of cardiac rehabilitation, such as Baduanjin exercise, on angina stability and frequency may also be related to improvements in the psychological state of patients.

In this study, some patients still had transient chest tightness and pain after coronary revascularization. Repeated chest tightness and pain after coronary revascularization will not only cause anxiety but may also cause doubt in terms of the doctors’ treatment recommendations, thus affecting patients’ compliance. In the early postoperative period, the patients’ condition gradually stabilized, and some chest tightness and pain symptoms improved spontaneously; however, some patients still suffered from recurrent or even aggravated symptoms. SAQ analysis showed that physical limitation and angina stability and frequency improved in both groups, with higher levels of angina stability in the Baduanjin exercise group, suggesting that Baduanjin exercise can improve the clinical symptoms of patients with AMI after PCI. The mechanisms may be due to the establishment of collateral circulation and improvement of blood and oxygen supply to cardiomyocytes brought about by postoperative rehabilitation training, recovery of cardiopulmonary function, and improvement of psychological state after standardized drug therapy. Nevertheless, the specific mechanism needs to be further verified.

This study has some limitations. First, although patients were supervised through WeChat and the patient exercise record sheet, it was still difficult to ensure compliance with the Baduanjin exercise program after discharge. Some patients could not complete the Baduanjin exercise twice daily as required. After persuasion, patient compliance improved; however, there was still a discrepancy between daily exercise volume and daily target exercise volume. Second, the standard of Baduanjin movement was difficult to unify. There are also additional limitations, such as the small sample size, limited source of cases, and short observation time, which need to be addressed in future studies. In future studies, more detailed exercise standards and graded exercise strategies for Baduanjin should be formulated. Thirdly, while we stipulated that participants should refrain from engaging in any aerobic exercises beyond the study specifications upon enrollment, maintain a consistent sleep pattern, and adhere to a low-salt and low-fat diet, we did not rigorously monitor their lifestyle post-discharge.

## 5. Conclusions

Baduanjin exercise for 8 weeks improved the cardiac function, exercise tolerance, psychological state, quality of life, and clinical symptoms of patients with AMI and anxiety and depression after PCI to a certain extent, but the long-term efficacy of such patients has not been confirmed. In clinical practice, less attention is paid to patients with anxiety and depression after PCI. Psychological counseling, exercise therapy, and drug treatment are of value in improving the body and mind. However, larger studies with longer follow-up are needed to confirm the effect.

## Acknowledgments

The authors wish to thank the patients for their cooperation regarding this publication. The authors also wish to thank Editage (www.editage.com) for English language editing.

## Author contributions

**Conceptualization:** Rong Li.

**Data curation:** Keyu Chen, Xiaoqin Chen.

**Investigation:** Liang Kang, Yihua Li, Qingmin Chu.

**Methodology:** Xinjun Zhao.

**Supervision:** Rong Li.

**Validation:** Qingmin Chu, Xinjun Zhao.

**Visualization:** Liang Kang, Yihua Li.

**Writing – original draft:** Liang Kang, Yihua Li.

## Supplementary Material


