# Supplementary material for: Effect of Baduanjin exercise on acute myocardial infarction in patients with anxiety and depression after percutaneous coronary intervention: A randomized controlled trial
Source: Medicine (Baltimore). 2024 Nov 8;103(45):e40225. doi: 10.1097/MD.0000000000040225 (PMC11557046; doi:10.1097/MD.0000000000040225)
Supplement: Supplementary file 1 [file medi-103-e40225-s001.docx]

**Supplemental Digital Content 1**

Kang *et al*. Effect of Baduanjin exercise on acute myocardial infarction in patients with anxiety and depression after percutaneous coronary intervention: a randomized controlled trial

#

**
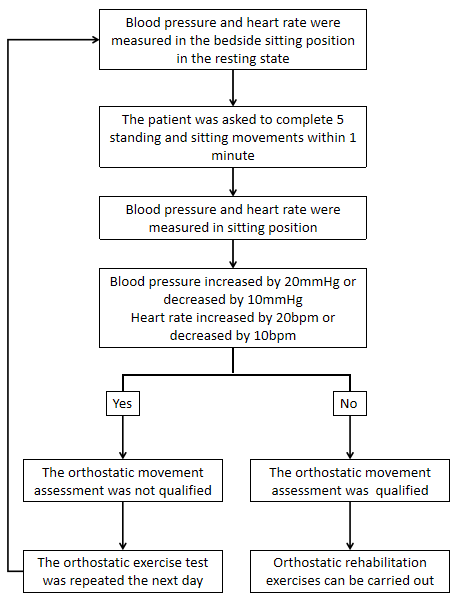
Fig. S1.** **Orthostatic exercise test
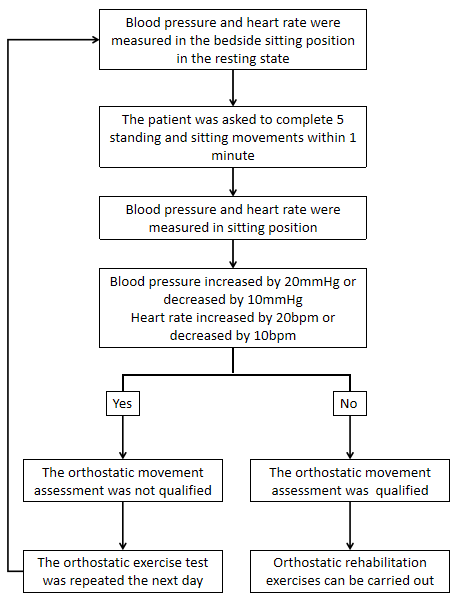
**
