# Supplementary material for: Effect of Baduanjin exercise on acute myocardial infarction in patients with anxiety and depression after percutaneous coronary intervention: A randomized controlled trial
Source: Medicine (Baltimore). 2024 Nov 8;103(45):e40225. doi: 10.1097/MD.0000000000040225 (PMC11557046; doi:10.1097/MD.0000000000040225)
Supplement: Supplementary file 2 [file medi-103-e40225-s002.docx]

**Supplemental Digital Content 2**

Kang *et al*. Effect of Baduanjin exercise on acute myocardial infarction in patients with anxiety and depression after percutaneous coronary intervention: a randomized controlled trial

#


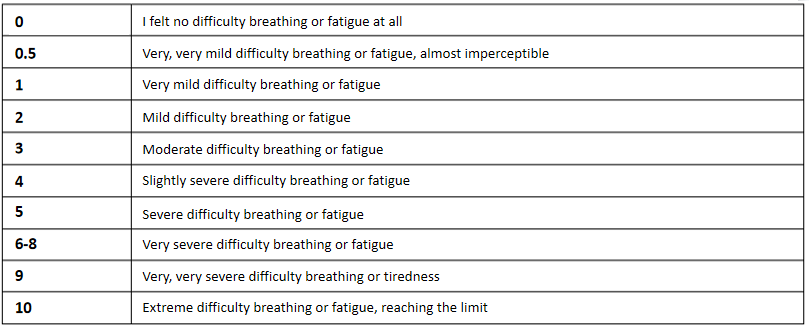


**Table S1. Borg scale**

**Supplemental Digital Content 3**

Kang *et al*. Effect of Baduanjin exercise on acute myocardial infarction in patients with anxiety and depression after percutaneous coronary intervention: a randomized controlled trial

#

| **Day(s)** | **Date** | **Exercise start time** | **Duration of exercise** | **Self**[**-evaluation**](javascript:;) | | | |
| --- | --- | --- | --- | --- | --- | --- | --- |
| **Week one** | | | | | | | |
| 1 |  |  |  | [excellent](javascript:;) | good | [ordinary](javascript:;) | poor |
| 2 |  |  |  | [excellent](javascript:;) | good | [ordinary](javascript:;) | poor |
| 3 |  |  |  | [excellent](javascript:;) | good | [ordinary](javascript:;) | poor |
| 4 |  |  |  | [excellent](javascript:;) | good | [ordinary](javascript:;) | poor |
| 5 |  |  |  | [excellent](javascript:;) | good | [ordinary](javascript:;) | poor |
| 6 |  |  |  | [excellent](javascript:;) | good | [ordinary](javascript:;) | poor |
| 7 |  |  |  | [excellent](javascript:;) | good | [ordinary](javascript:;) | poor |
| **Week two** | | | | | | | |
| 1 |  |  |  | [excellent](javascript:;) | good | [ordinary](javascript:;) | poor |
| 2 |  |  |  | [excellent](javascript:;) | good | [ordinary](javascript:;) | poor |
| 3 |  |  |  | [excellent](javascript:;) | good | [ordinary](javascript:;) | poor |
| 4 |  |  |  | [excellent](javascript:;) | good | [ordinary](javascript:;) | poor |
| 5 |  |  |  | [excellent](javascript:;) | good | [ordinary](javascript:;) | poor |
| 6 |  |  |  | [excellent](javascript:;) | good | [ordinary](javascript:;) | poor |
| 7 |  |  |  | [excellent](javascript:;) | good | [ordinary](javascript:;) | poor |

| **Day(s)** | **Date** | **Exercise start time** | **Duration of exercise** | **Self**[**-evaluation**](javascript:;) | | | |
| --- | --- | --- | --- | --- | --- | --- | --- |
| **Week three** | | | | | | | |
| 1 |  |  |  | [excellent](javascript:;) | good | [ordinary](javascript:;) | poor |
| 2 |  |  |  | [excellent](javascript:;) | good | [ordinary](javascript:;) | poor |
| 3 |  |  |  | [excellent](javascript:;) | good | [ordinary](javascript:;) | poor |
| 4 |  |  |  | [excellent](javascript:;) | good | [ordinary](javascript:;) | poor |
| 5 |  |  |  | [excellent](javascript:;) | good | [ordinary](javascript:;) | poor |
| 6 |  |  |  | [excellent](javascript:;) | good | [ordinary](javascript:;) | poor |
| 7 |  |  |  | [excellent](javascript:;) | good | [ordinary](javascript:;) | poor |
| **Week four** | | | | | | | |
| 1 |  |  |  | [excellent](javascript:;) | good | [ordinary](javascript:;) | poor |
| 2 |  |  |  | [excellent](javascript:;) | good | [ordinary](javascript:;) | poor |
| 3 |  |  |  | [excellent](javascript:;) | good | [ordinary](javascript:;) | poor |
| 4 |  |  |  | [excellent](javascript:;) | good | [ordinary](javascript:;) | poor |
| 5 |  |  |  | [excellent](javascript:;) | good | [ordinary](javascript:;) | poor |
| 6 |  |  |  | [excellent](javascript:;) | good | [ordinary](javascript:;) | poor |
| 7 |  |  |  | [excellent](javascript:;) | good | [ordinary](javascript:;) | poor |

| **Day(s)** | **Date** | **Exercise start time** | **Duration of exercise** | **Self**[**-evaluation**](javascript:;) | | | |
| --- | --- | --- | --- | --- | --- | --- | --- |
| **Week five** | | | | | | | |
| 1 |  |  |  | [excellent](javascript:;) | good | [ordinary](javascript:;) | poor |
| 2 |  |  |  | [excellent](javascript:;) | good | [ordinary](javascript:;) | poor |
| 3 |  |  |  | [excellent](javascript:;) | good | [ordinary](javascript:;) | poor |
| 4 |  |  |  | [excellent](javascript:;) | good | [ordinary](javascript:;) | poor |
| 5 |  |  |  | [excellent](javascript:;) | good | [ordinary](javascript:;) | poor |
| 6 |  |  |  | [excellent](javascript:;) | good | [ordinary](javascript:;) | poor |
| 7 |  |  |  | [excellent](javascript:;) | good | [ordinary](javascript:;) | poor |
| **Week six** | | | | | | | |
| 1 |  |  |  | [excellent](javascript:;) | good | [ordinary](javascript:;) | poor |
| 2 |  |  |  | [excellent](javascript:;) | good | [ordinary](javascript:;) | poor |
| 3 |  |  |  | [excellent](javascript:;) | good | [ordinary](javascript:;) | poor |
| 4 |  |  |  | [excellent](javascript:;) | good | [ordinary](javascript:;) | poor |
| 5 |  |  |  | [excellent](javascript:;) | good | [ordinary](javascript:;) | poor |
| 6 |  |  |  | [excellent](javascript:;) | good | [ordinary](javascript:;) | poor |
| 7 |  |  |  | [excellent](javascript:;) | good | [ordinary](javascript:;) | poor |
| **Day(s)** | **Date** | **Exercise start time** | **Duration of exercise** | **Self**[**-evaluation**](javascript:;) | | | |
| **Week seven** | | | | | | | |
| 1 |  |  |  | [excellent](javascript:;) | good | [ordinary](javascript:;) | poor |
| 2 |  |  |  | [excellent](javascript:;) | good | [ordinary](javascript:;) | poor |
| 3 |  |  |  | [excellent](javascript:;) | good | [ordinary](javascript:;) | poor |
| 4 |  |  |  | [excellent](javascript:;) | good | [ordinary](javascript:;) | poor |
| 5 |  |  |  | [excellent](javascript:;) | good | [ordinary](javascript:;) | poor |
| 6 |  |  |  | [excellent](javascript:;) | good | [ordinary](javascript:;) | poor |
| 7 |  |  |  | [excellent](javascript:;) | good | [ordinary](javascript:;) | poor |
| **Week eight** | | | | | | | |
| 1 |  |  |  | [excellent](javascript:;) | good | [ordinary](javascript:;) | poor |
| 2 |  |  |  | [excellent](javascript:;) | good | [ordinary](javascript:;) | poor |
| 3 |  |  |  | [excellent](javascript:;) | good | [ordinary](javascript:;) | poor |
| 4 |  |  |  | [excellent](javascript:;) | good | [ordinary](javascript:;) | poor |
| 5 |  |  |  | [excellent](javascript:;) | good | [ordinary](javascript:;) | poor |
| 6 |  |  |  | [excellent](javascript:;) | good | [ordinary](javascript:;) | poor |
| 7 |  |  |  | [excellent](javascript:;) | good | [ordinary](javascript:;) | poor |

**Table S2. Patient exercise record sheet**
